# Strategies for the Production of Molecular Animations

**DOI:** 10.3389/fbinf.2022.793914

**Published:** 2022-05-16

**Authors:** Erik Werner

**Affiliations:** RNS Berlin, Berlin, Germany

**Keywords:** molecular animation, scientific visualisation, consistent complexity, design, advance the audience, cinematic storytelling, molecule motion, dynamic realism

## Abstract

Molecular animations play an increasing role in scientific visualisation and science communication. They engage viewers through non-fictional, documentary type storytelling and aim at advancing the audience. Every scene of a molecular animation is to be designed to secure clarity. To achieve this, knowledge on design principles from various design fields is essential. The relevant principles help to draw attention, guide the eye, establish relationships, convey dynamics and/or trigger a reaction. The tools of general graphic design are used to compose a signature frame, those of cinematic storytelling and user interface design to choreograph the relative movement of characters and cameras. Clarity in a scientific visualisation is reached by simplification and abstraction where the choice of the adequate representation is of great importance. A large set of illustration styles is available to chose the appropriate detail level but they are constrained by the availability of experimental data. For a high-quality molecular animation, data from different sources can be integrated, even filling the structural gaps to show a complete picture of the native biological situation. For maintaining scientific authenticity it is good practice to mark use of artistic licence which ensures transparency and accountability. The design of motion requires knowledge from molecule kinetics and kinematics. With biological macromolecules, four types of motion are most relevant: thermal motion, small and large conformational changes and Brownian motion. The principles of dynamic realism should be respected as well as the circumstances given in the crowded cellular environment. Ultimately, consistent complexity is proposed as overarching principle for the production of molecular animations and should be achieved between communication objective and abstraction/simplification, audience expertise and scientific complexity, experiment and representation, characters and environment as well as structure and motion representation.

## 1 Introduction

Modern technology makes video an easily accessible and therefore omnipresent medium in our lives and therefore also in the fields of knowledge and communication where it unfolds its full potential in the form of molecular animations. Molecular animation can be described as motion design for biological macromolecules. Since these nano-scale characters are not directly visible to the human eye, we need to draw on an array of visualisation methods to communicate them. Combining scientific illustration with motion gives us the opportunity to visualise the dynamics of the molecular system.

Molecular animations can be used in science communication, education and research (Iwasa, 2010 and 2015). They create interest, increase memory and lead to better comprehension of complex subjects. In research, they provide researchers an insight into processes by summarising and contextualising a mechanism and can also support grant applications, marketing, social and environmental campaigns and many more.

In 2008 Gael McGill announced “Molecular Movies … Coming to a Lecture near You” (McGill, 2008), describing the upcoming trend to use professional 3D software known from movies. In 2010 a group of experts met at the Workshop on Molecular Animation in San Francisco (Bromberg, Chiu and Ferrin, 2010) to discuss needs and requirements. Since then, several major publications appeared focused on scientific visualisation (O’Donoghue et al., 2010a; O’Donoghue et al., 2010b; Johnson and Hertig, 2014; Kozlíková et al., 2017; Goodsell and Jenkinson, 2018; Olson, 2018) or visualisation software (Goddard and Ferrin, 2007; Martinez et al., 2019). In addition, the potential of animations has been highlighted by the exemplary work of Iwasa (Iwasa, 2010 and 2015), Berry (TED, 2012) and others, and software development, for example of ePMV (Johnson et al., 2011) and Molecular Maya (McGill, 2010), enable the use of structural data in professional 3D software.

This perspective article formulates a number of guidelines with relevance for molecular animations based on knowledge and literature from the main fields design, scientific visualisation, molecular kinetics/kinematics and cinematography/storytelling. It may contribute to a theoretical basis for the field of scientific and especially molecular animation.

Molecular animation is understood here as the visualisation of the structure and dynamic of macromolecular biomolecules and their substrates within the context of the living cell, at the molecular nano-scale. Molecular animation therefore can be seen as a subspace of data visualisation (with structural and related dynamic data being a subset of all scientific data) and medical illustration/animation, that includes the biological mesoscale (larger than molecular complexes, smaller than a cell; see Johnson (VIZBI, 2012b), Le Muzic et al. (2014) and Goodsell et al. (2020) for details) and macroscale dimensions (cells, organelles, organs, organisms). Consequently, this article concentrates on aspects and principles most relevant to molecular animations and may omit some others. Please see the Supplement for a detailed description of the methodology used in deriving these guidelines.

## 2 Advance the Audience and Engage It Through Storytelling

### 2.1 Build the Basics and Advance the Audience

Every design object should serve a purpose that benefits the user. A molecular animation is an audiovisual design object, usually aimed at a specific target audience whose expertise level may vary; see McGill (VIZBI, 2012a) and Johnson and Hertig (2014). For an audience to benefit from an animation, it is essential to adjust the complexity to the actual expertise level. So it is deemed a good idea to introduce a topic with basic knowledge and allow everybody to connect, irrespective of their expertise. The higher the audience’s expertise level and audiovisual literacy the shorter the introduction can be. An animation may quickly go into the latest results and very complex detail when it addresses advanced experts. However, it may still be necessary to explain the basic principles, the relevant visual conventions and also to refresh the memory of a viewer.

The audience generally engages with the animation to learn something new and interesting. It should therefore be the goal of every animation to advance the audience - to introduce something new, more complex, more challenging - and allow them to extend their knowledge (Johnson and Hertig, 2014). This means that the complexity level of the animation can go at least one step further than the one that is indicated by the expertise limit of the typical viewer.

### 2.2 Adjust the Video Output to Reflect the Consumption Scenario

A user consumes a molecular animation through a digital screen, either in a guided presentation or in a stand-alone format, for example on a video platform, which directly influences the time of engagement (Frankel and DePace, 2012). In a presentation format, a speaker usually guides the audience through the animation within a larger context adjusted for the specific expertise level of the actual audience. The animation is usually shown only once and therefore needs to put special emphasis on clarity and simplicity. A stand-alone animation can be paused and repeated and therefore allows more complexity. The content of an animation can be adjusted to those different scenarios through an output strategy that makes use of a modular toolbox and animation helpers such as labels, sound, voiceover, subtitles or annotations. A full parent version includes all available scenes and covers all relevant communication objectives. For a specific scenario, a selection of scenes serves as a derivative.

### 2.3 Use Filmmaking Production Techniques

The output strategy for an animation should be planned at the very beginning of the production, a process that follows the three production stages similar to a movie; please see Sharpe et al. (2008), Jantzen et al. (2015) and Lepito (2018) for details. The pre-prodcution stage includes the agreement on the communication objectives, decisions on a look and feel (style, colour, typography, narration, etc.), discovery of the story, scripting, storyboarding, creation of animatics and the output strategy. The production phase includes the creation of models and their dynamic animation as well as the implementation of lighting, cameras, materials, textures and shaders to create a render of each frame. In the post-production stage, individual sequences are combined into a composite, combined into a final edit with labels and sound and finally rendered out in a delivery format. While a molecular movie requires special knowledge mainly in the first two stages, post-production does not fundamentally differ from most video or movie projects. Subsequent chapters therefore concentrate on the preparation and production of molecular movies.

### 2.4 Engage the Audience Through Storytelling

A typical research project is structured as a chronological sequence of concept/hypothesis development, planning, experiment, data analysis and data interpretation. However, many research reports already use narrative elements in the IMRAD format to present the knowledge: introduction (exposition), methods (rising action), results (climax), analysis (falling action) and discussion (resolution) (ElShafie, 2018). In an animation, we can make use of a narrative. Storytelling can attract the audience’s attention, make them care and leave a lasting impression by including stakes and allowing the audience to relate to the story (Ma et al., 2012; Lepito, 2018). This makes the science more meaningful to them without compromising on scientific accuracy, objectivity and therefore credibility (ElShafie, 2018). Due to the persuasive nature of narratives, science animators need to include ethical considerations related to the underlying communication objective (persuasion or comprehension), the level of accuracy (external realism, representativeness) or the use of narrative at all, especially when addressing non-expert audiences (Dahlstrom, 2014).

**FIGURE 1:**
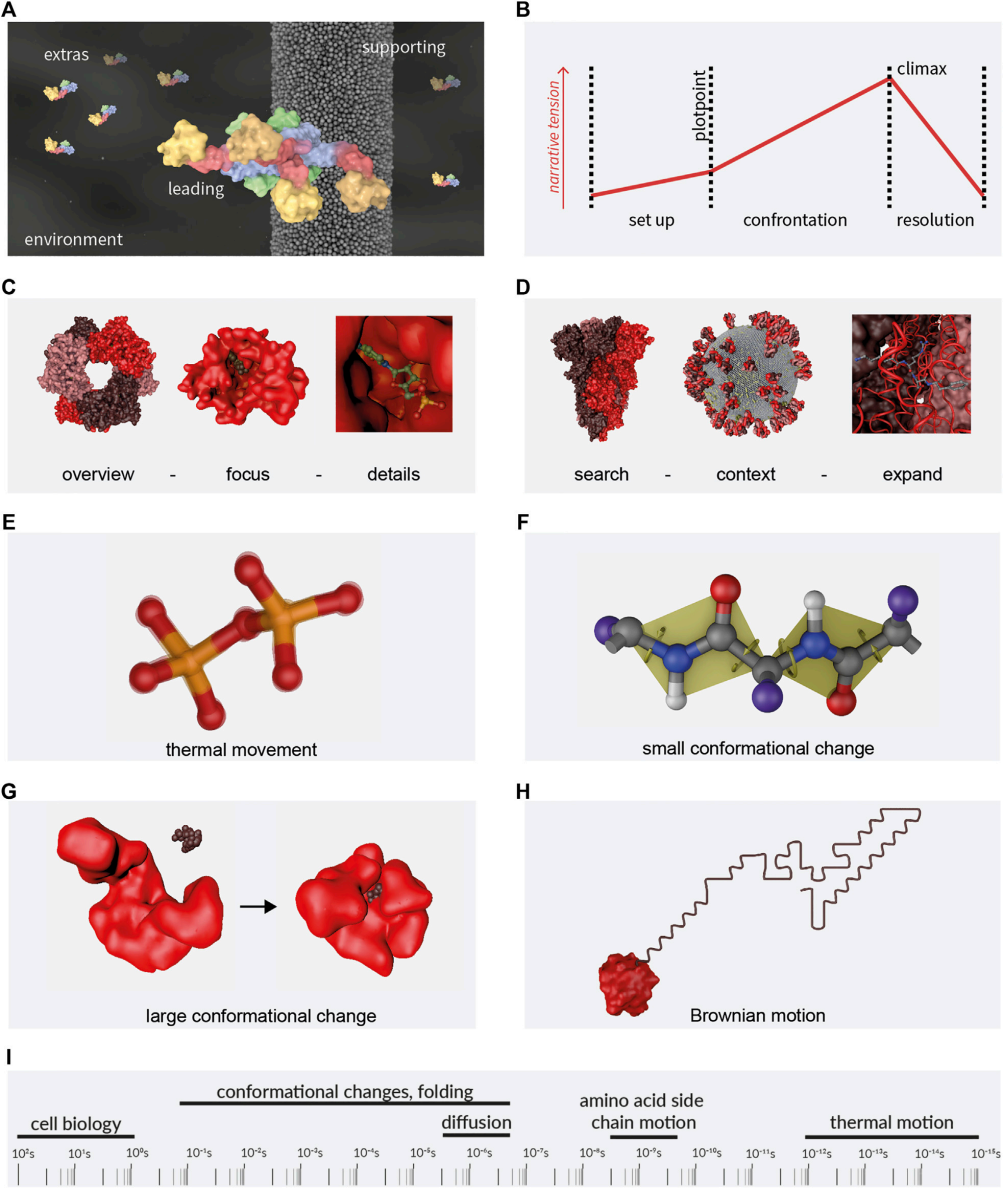
Storytelling and motion. **(A)** Molecular Actors. **(B)** Molecular Storytelling. The three stages of a classical story. Presentation Order, **(C)** overview first (hexamer in the example), zoom (monomer with ligand) and filter (ligand details); and **(D)** search (spike protein), show context (whole virus), expand (detail of the spike protein hinge region). Visualisation of the movement in/of macromolecules: **(E)** thermal movement **(F)** small and **(G)** large conformational changes and **(H)** Brownian motion. **(I)** Timescales of biological processes. Molecules depicted are: **(A)** Dynamin1, PDB code 3SNH, (Faelber et al., 2011); **(C)** and **(H)** NMNAT1, PDB code 1GZU (Werner et al., 2002); **(D)** SARS-CoV-2 proteins S [PDB code 1KDI, (Gobeil et al., 2021)], E [PDB code 7K3G (Mandala et al., 2020)], M by Mahtarin et al. (2020) and Swiss-Model entries for P0DTC4; **(G)** Adenylat Kinase, PDB codes 4AKE (Müller et al., 1996) and 2ECK (Berry et al., 2006). PDB: Protein Database (Berman et al., 2000); Swiss-Model Repository (Bienert et al., 2017).

The cinematic genre of molecular animations is best described as non-fictional documentary. Documentary storytellers must not invent and cannot make compromises when it comes to the facts. Instead, they need to be guided by and find the story in the material itself (Bernard, 2010). The story itself may be one of exploration, where the researchers are portrayed on their journey to discovery (Berlin, 2016). This is comparable to the well known narrative of the hero’s journey (Vogler, 2007). However, the molecular story may as well stand on its own. The molecular characters in an animation can be seen as playing roles similar to actors in a movie, even though they do not make conscious decisions but rather follow the laws of physics. Main characters carry the story, side characters support it and extras create the background, see Figure 1A for an example. All of them act in an environment that can have a strong influence on the story by setting the location and external conditions.

Contextualised in the wider field of data visualisation, a molecular animation belongs to one of seven genres of narrative visualisation: film/video/animation. The narrative structure tactics is strongly author-driven. As such, an animation is characterised by linear ordering of scenes, heavy use of labels, headlines and annotations (messaging) and a lack of interactivity (Segel and Heer, 2010). The author is in full control of the animation which constitutes passive storytelling (Wolhfart and Hauser, 2007).

### 2.5 Chose the Story Structure

Any story can be characterised by the three act structure that goes back to Aristotle’s Poetics (see the english translation, (Aristotle, 1996)) and Figure 1B) and includes a setting (establishment of environment and characters; act 1), a plot with rising narrative tension (act 2, the protagonist on a pursuit) and a resolution (act 3), see also ElShafie (2018). The structure of the story however does not have to be linear. It is determined by the tools of cinematography, editing and compositing. A viewer can get a certain understanding of the topic through an overview that shows all involved elements at the same time. It helps creating a reliable and recognisable framework to come back to when needed. While zooming into detail, the content is filtered and unnecessary element and details are left out. This follows Ben Shneidermann’s visual information-seeking mantra “overview first, zoom and filter, then details on demand” (Shneiderman, 1996) (Figure 1C). For datasets with high complexity, an alternative is: “search, show context, expand”, where we begin with a starting point, reflect on the contextual aspects and expand further context and detail when needed (Figure 1D; Munzner, 2014). Other story structures include comparative visualisation (side-by-side comparison) and iterative visualisation (a repetitive pattern when focusing on several features in the same context), see Wohlfart and Hauser (2007) and Ma et al. (2012).

## 3 Design Every Scene to Secure Clarity

Many molecular animation concepts include a storyboard with illustrated signature frames, the narration and possibly animatics for the timing. The more complex a topic and an animation are, the more important a storyboard becomes. Every signature frame, the transitions between them and the relative movement within a scene need to be designed to achieve the specific communication objective of that scene. Technically, this can be achieved by keyframe interpolation, particle or molecular dynamics with defined starting points and dynamic field parameters.

### 3.1 Follow Design Principles

The signature frames are individual images that represent important situations of the story. The molecular animator should be able to create a clear design for them and therefore have a good knowledge on design principles from various fields, most importantly graphic design, motion design, user interface design and cinematography/film. The design principles can be categorised into five (partially overlapping) areas: draw attention, guide the eye, establish relationships, convey dynamics and create emotion /reaction. The methodology for the selection of principles relevant for molecular animations is described in the Supplement, including a visualisation of the principles in Supplementary Figures 9S–13S; those from general graphic design are mainly based on monographs “Graphik und Gestaltung” (Wäger, 2014) and “Perception of Design” (Ware, 2012).

### 3.2 Use the Tools of Cinematography

An animation is created by moving from signature frame to signature frame and includes the relative movement of characters and camera view. Characters may enter, stay in or leave the frame. Or they can move with the camera relative to other characters or the environment. Also, camera movement can be combined with character movement. The techniques and principles of non-dialogue cinematic storytelling (Sijll, 2005; Mercado, 2010; Raschke, 2013) help to reach the communication objectives and include setting the look and feel through composition and lighting and finally positioning and moving the camera through a scene while maintaining that look and feel. The attention of the audience, its emotions and interest are led by changing these parameters and also the sound design. Viewing axes and depth of field establish the relation between characters and both character and camera motion establish an order of events. The choice of focal length, editing, transitions and time altercations all play important roles and are chosen dependent of the communication objective. Overreaching principles from cinematographic storytelling include “story is king”, were all elements visible on the screen support the story and “show, not just tell” where the eye is guided by visual highlighting (Lepito, 2018).

### 3.3 Learn From User Interface Design

The motion lessons of interface design (material.io/Google, 2021) support the choreography of character movements. They deal with the speed of incoming and outgoing elements, their duration in the frame and the grouping of movements based on the complexity. This way, we can define the movement path or the fade-in/fade-out properties of the characters.

### 3.4 Ignore the “Disney Animation Principles”

It needs to be mentioned, that the well known set of “Disney animation principles” (Thomas and Johnston, 1981) does not apply to the nano-world of molecules. They cover *timing and spacing, easing, mass and weight, squash and stretch, follow through and overlapping, secondary action, arcs, solid drawing, anticipation, exaggeration, staging and appeal*. With the help of those principles, natural movements are recreated based on material properties, following the laws of classical physics and building on every-day-life experience in the macro world. An experience that does not exist in the nano-world of atoms and molecules. Here, movement is determined by random collisions, diffusion gradients and thermal motion. So, with the exception of the more general principles *staging* and *appeal*, those for the design of motion need to be set aside for the animations of molecules.

## 4 Chose Adequate Representation to Illustrate Current Knowledge

### 4.1 Simplify and Abstract

Clarity is the overall goal of any design process and it is therefore also important for scientific visualisation in general. It is often reached by simplification (displaying fewer items) and abstraction (using simpler forms of an item), or both in combination. Clarity avoids clutter in the frame while unburdening the perceptual system of the viewer. However, it needs to be carefully balanced with the addition of more complex detail in order to advance the audience. In fact, in the nano-world of macromolecules, there is always a certain level of abstraction involved in scientific visualisations which automatically leaves room for interpretation (Sharpe et al., 2008). A taxonomy of types of abstraction includes symbolic representations, schematic diagrams, graphs, cartoons and realistic representations (Offerdahl, et al., 2018; Goodsell and Jenkinson, 2018). They all can play a role in molecular animations and need to be chosen dependent on the audience expertise level and the specific communication objective.

### 4.2 Chose a Representation of Biological Macromolecules

**FIGURE 2:**
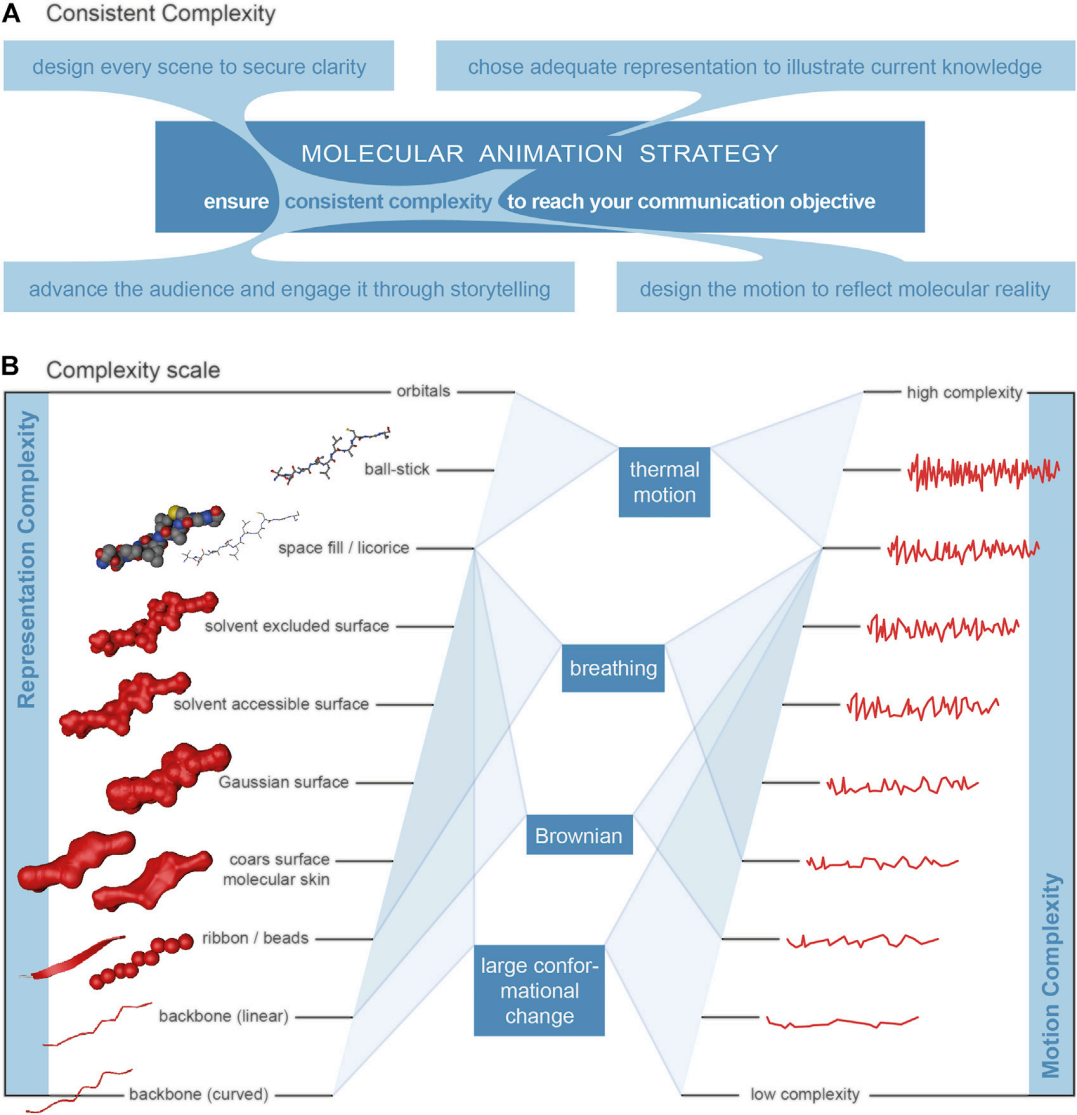
Complexity. **(A)** Visualisation of the overarching principle for the production of molecular animations, connecting the four main elements audience, design, representation and motion. **(B)** Relationship between the level of detail of a macromolecule representation and the motion complexity for four relevant types of motion. The complexity of motion (on the right) is visualised by a waveform-like line where a higher frequency represents more frequent changes and therefore higher complexity. Representations, level of detail (left). Selected representations of a beta-sheet poly-peptide on a scale of increasing level of detail/complexity from the bottom to the top. Illustrative abstract representations: curved backbone, linear backbone and ribbon/cartoon (Richardson, 1985; Carson, 1987). Surface abstractions: beads and coarse approximation (Blinn, 1982). Atomistic surface representation: molecular skin surface (MSS), Gaussian surface, solvent accessible surface (SAS) (Sanner, et al., 1996) and solvent excluded surface (SES) (Connolly, 1983). Atomistic space-filling representation: space-fill (Corey and Pauling, 1953; Koltun, 1965a and; Koltun, 1965b). Atomistic bond-centric representations: licorice, ball-and-stick (Fieser, 1963).

The visualisation of a biological macromolecule (molecular graphics) can range from very simple to very complex. Illustrative and abstract representations include 1D formats (letter codes), 2D formats (letter-code with crosslinks, schematic) and 3D formats (arbitrary organic shapes, backbone and ribbon/cartoon representations). 3D-surface abstractions include beads representations (one bead per subunit, e.g., amino acids) and coarse approximation. 3D atomistic surface models show more detail and include convolution surface models (e.g., Gaussian), molecular skin surfaces, ligand excluded surfaces, solvent excluded surfaces and solvent accessible surfaces. 3D atomistic space filling models are characterised by each atom being represented by a sphere with a radius based on the Van der Waals radii. 3D atomistic bond-centric models include hyperballs, licorice and ball-and-stick representations or even quantum mechanical models. Please see Kozlíková et al. (2017), Goodsell and Jenkinson (2018) and Olson (2018) for detailed descriptions and Johnson and Hertig (J2014), Biocinematics (2016) and O'Donoghue et al. (2010b) for overviews. Figure 2B includes the representations of a short beta-strand polypeptide with increasing complexity from bottom to top.

### 4.3 Respect Constraints

Choosing the adequate representation can be challenging and depends on several factors like the actual, specific communication objective, the experimental data available, the topical context, the target audience expertise level, and others. To avoid misconceptions, it is recommended to avoid causing superficial understanding due to over-simplification and abstraction. A depiction may be taken literally and not interpreted according to underlaying scientific knowledge. Whole biological concepts can be basically understood like this, but actual insight may not go beyond the simplicity of the representation explaining it (Goodsell and Jenkinson, 2018). Mixed representations are popular to highlight details and help to establish the character relationships.

### 4.4 Ensure Scientific Authenticity and Transparency

At the same time it should also be avoided to imply more knowledge than the data actually provides. The quality and resolution of the available experimental data restricts the level of structural detail shown in a scientific visualisation or animation. However, that does not keep us from integrating data from all kinds of resources or even models, predictions or hypotheses (O’Donoghue et al., 2010b; Ward et al., 2013). Even with gaps in the data, it may still be useful to display the complete macromolecule and to model the structure gaps based on the best knowledge available. We always should consider full length, native proteins for a more realistic visualisation because it reflects the natural cellular environment. This use of artistic licence plays an important role in scientific visualisation; see Goodsell and Jenkinson (2018) and Goodsell and Johnson (2007) for details on this topic. In the spirit of scientific authenticity - which is fundamental for the credibility of molecular animations - it is good practice to mark the use of artistic licence by differentiated representation, render style, colour and/or by annotation. This ensures transparency and therefore increases the accountability of an animation (Jantzen et al., 2015). The viewer should be able to judge, which aspects are data derived and which are more hypothetical.

## 5 Design the Motion to Reflect Molecular Reality

The design of motion requires biophysical knowledge from molecule kinetics and kinematics. For biological macromolecules four types of motion are most relevant: thermal motion, small and large conformational changes (all intrinsic) and Brownian motion (with a molecule as one unit), see visualisations in Figures 1E–H. More specific cases are the two-dimensional movement of a protein in a membrane and directed motor activities like the kinesin or myosin transport activities. Please see Johnson and Hertig (2014) and O’Donoghue et al. (2010b) for detail and Phillips et al. (2012), Nelson (2014) and Kuriyan et al. (2012) for the biophysical principles. It is a massive challenge to reflect various molecular motion time scales that span 17 orders of magnitude (McGill, 2008), see Figure 1I. However, the amplitude of a movement is often proportional to its frequency, so very rapid movements can be left out when the complete protein is shown. They need to be considered though, when the intrinsic dynamic plays a role for the function (Eisenmesser et al., 2005).

### 5.1 Thermal Motion

Thermal motion is observed for any atom in a molecule and has a very high frequency, dependent on the temperature. The higher the temperature, the stronger the positional dislocation. It can often be neglected in an animation, especially when other, lower frequency motions are shown. It remains relevant in very complex and highly detailed animations, where visualising the movement of individual atoms or even quantum mechanical detail (orbitals) increases the accuracy of the representation.

### 5.2 Small Conformational Changes

Small conformational changes are based on the rotational freedom of a bond between two atoms, e.g., the rotation between peptide bonds (see Dong, 2021). Accumulated along a network of macromolecule residues, they can add up to far-reaching and larger movements. The rotational freedom can be restricted through non-covalent bonds like hydrogen-bonds, salt-bridges or hydrophobic interactions. Small conformational changes should be included in animations with high complexity and especially when they are central for the visualisation of the molecular mechanism and therefore the function of the macromolecule. Together with thermal motion, small conformational changes are responsible for the “breathing” of a protein (Makowski et al., 2008) which can be represented by a fluctuating surface.

### 5.3 Large Conformational Changes

Biological macromolecules and especially proteins often have domains, subdomains or other structural motifs (like alpha-helices and beta-sheets) that show a certain rigidity within themselves while flexible loops between them are responsible for movements of those substructures relative to each other. Flexible loops themselves also may undergo large conformational changes to fulfil a function. Many biological processes depend on this type of large conformational changes. They are often visualised by the intrinsic movement of surface representations, but also ribbon-type cartoons.

### 5.4 Brownian Motion

When macromolecules in a cellular environment move as one unit, they usually do so by random collision with other molecules, caused by their thermal motion and often described as random walk. Collisions create an external force that is not directional, so the movement of the macromolecule is random and not caused by long-range attracting forces between two reaction partners. This provides a challenge for a molecular animator, who needs to find a balance between the visualisation of the non-directional nature of the random motion and the actual approach of reaction partners within the timeframe of the scene. Le Muzic et al. (2014) describe a way to blend random walk with linear interpolation in a particle based metabolic network model to simulate this motion.

### 5.5 Ensure Dynamic Realism

The motion-equivalent to structural detail in a representation is called dynamic realism by Jantzen (Biocinematics, 2016). Both, structural and dynamic information need to be considered in a molecular animation where the representation becomes unrealistic when it is inconsistent between structure and motion. Dynamic realism means that abstract, less detailed structure representations go along with simple dynamics and more detailed structure representations also require more realistic dynamic representations (Biocinematics, 2016). The complexity of motion is interpreted here as the changes of direction and acceleration, rather than those of the actual speed.

### 5.6 Reflect a Crowded Cellular Environment

In a crowded cellular environment, the set of principles described by Jantzen et al. (2017) should be respected. In short and partially merged: I. permanent Brownian motion causes collisions and therefore movement, there are no long-range forces; II. biological macromolecules underlay internal flexibility but they have defined boundaries; III. in the cell, there are many instances of a molecule and not all react; IV. the cell is a crowded environment that does not show aqueous effects. The representation of individual elements in a crowded environment however does not require the full detail of all elements. The further away an element from the main focus, the fewer atoms can be displayed without losing any major information (Le Muzic et al., 2014, 2015).

## 6 Discussion

In systems of high dynamics such as the nano-world of biological macromolecules, the medium of video can play out its strength. Compared to explanatory text, an animation can often be more efficient and intuitive. Compared to a diagram or illustration it can be more accurate and detailed. Consequently, an animation used in research and education should reflect the comprehensive knowledge of a system. Only then the complexity of the system can be communicated realistically and used for the development and evaluation of hypotheses. Failure to reflect the complexity leads to the misconception that a complex system is indeed simple and also to flawed future experiment design. As a consequence, it is recommended to use 2D sketches and cartoon style shading when little is known about a system and a 3D animation for established mechanisms (Iwasa, 2010).

### 6.1 Consistent Complexity

The main determinant for the complexity of a molecular animation is the complexity of the actual communication objective, the point that needs to come across, the focus of attention. The communication objective of a particular scene can be very specific and is usually related to one of these two categories (or a combination of both): a) the properties of the components - structure, chemical and physical properties, relation towards each other, etc.; and b) the dynamic of the system, the changes over time. The complexity of a communication objective is then directly associated to the complexity of the main element. The principle of consistent complexity (see Figure 2A for a visualisation) is proposed as overarching principle for the production of molecular animations. The communication objectives can be reached with clarity when consistent complexity is achieved for the relevant aspects.

#### 6.1.1 Communication Objective and Abstraction/Simplification

A simple point is often made best with a simple rather than a complex visualisation, as the latter can distract or overwhelm the viewer. A complex point however usually requires a more complex visualisation because the detail is just not there in a simpler representation and the viewer is usually not able to interpret it on his/her own.

#### 6.1.2 Audience Expertise and Scientific Complexity

An animation should aim towards advancing the audience but not overwhelm it. Hence, the scientific complexity of the visual story needs to be in balance with the audience’s level of expertise and visual literacy. The theoretical framework for visual storytelling developed by Botsis et al. (2020) is helpful to evaluate the individual characteristics of a visual story. It goes back to Cairo’s Visualization Wheel (Cairo, 2012) and comprises six contrasting pairs of characteristics: conceptualisation - figuration, functionality - decoration, density - lightness, multidimensionality - unidimensionality, originality - familiarity and novelty - redundancy. A consistent story represents the set of characteristics that are mentioned first in a pair (high complexity) or second (low complexity). Practically, a modular approach for the combination of scenes can help to target a specific audience.

#### 6.1.3 Experiment and Representation

The representation detail should match the quality and resolution of the existing experimental basis. This helps to avoid the impression of more knowledge than there actually is. For scientific authenticity, the use of artistic licence should be transparently annotated but not avoided if it helps the representation of the realistic conditions.

#### 6.1.4 Characters and Environment

Large differences in the representation complexity of neighbouring character levels (main, side, extras, environment) should be avoided. This is an issue for mixed representations. A more gradual change of the level of detail helps to avoid visual breaks. The environment should have less complexity than the characters, but may well become the focus of attention for another communication objective and have its complexity increased for another scene.

#### 6.1.5 Structure and Motion Representation

The complexity of the structure representation needs to be matched with the one of the motion representation (dynamic realism). While the complexity of a structure representation is easily understood as the level of detail, the complexity of a motion is less well intuitive. Figure 2B suggests a complexity scale for properties and their associated movements. The level of detail of a biomolecule representation does not necessarily correlate with the speed and amplitudes of the different types of motion.

We need to look at the different motion types in order to relate the complexity of a representation with the complexity of a motion. Figure 2B sets them into relation and gives an indication which type of motion should be shown in association with a certain representation detail. Thermal motion should be included in an animation when the communication objective focusses on the atomistic reaction detail. It can be neglected at protein (surface) level, where protein breathing should be included and thermal motion adds next to nothing to the accumulative motion. Large conformational changes are often at the heart of an animation and the centre of the communication objective. The inclusion in the animation is therefore a matter of course and relevant on the domain/subdomain level. Brownian Motion is relevant for the overall motion of molecules as a unit and enables reactions between molecules in the first place. It should therefore be included on that protein level. However, it needs to be mentioned that the relations described are a first indication only and that a specific communication objective may well require different combinations of representation and motion complexity.
