# Supplementary material for: Strategies for the Production of Molecular Animations
Source: Front Bioinform. 2022 May 16;2:793914. doi: 10.3389/fbinf.2022.793914 (PMC9580893; doi:10.3389/fbinf.2022.793914)
Supplement: Supplementary file 1 [file DataSheet1.pdf]

Supplement: Principles relevant for the production of molecular animations - methodology, analysis and results

An expanded version of this work is available (Werner, 2019). The reference list can be found in the main article.

Compatibility of design principles: Methodology

The design principles are categorised and investigated according to their compatibility with scientific visualisation principles. To do this, each principle is analysed with regard to its relevance for still images or motion pictures and placed on a vertical axis. The relevance in the macro or nano-world is added on a horizontal axis. On top, principles are categorised by the objective of the principle via the symbol and their applicability to the 2D- or 3D-world through the colour of the symbol. Please see Figure S1 for a general graph (a) and the legend (b). With "objective of the principle", the following categories are meant:

- attention - the principle describes a rule that draws attention;
- eye - the item described in the principle guides the eye;
- dynamic - the principles covers a topic that conveys dynamic;
- relationship - the principle establishes a relationship between items in the frame/ scene;
- goal - the principle describes a method to trigger emotions or a reaction, dependent on the communication objective.

(a) (b)

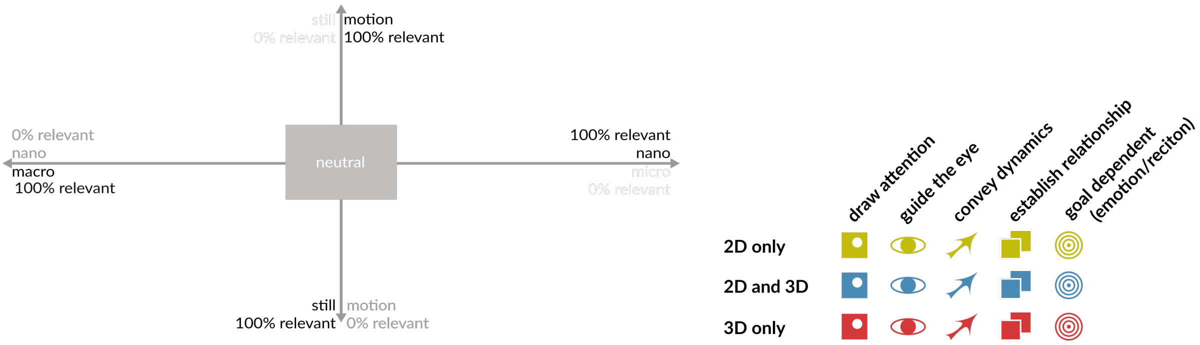

Figure S1: (a) Categorisation template and (b) legend.

Results and analysis: General Aspects of Design *(Wäger, 2014:25ff)*

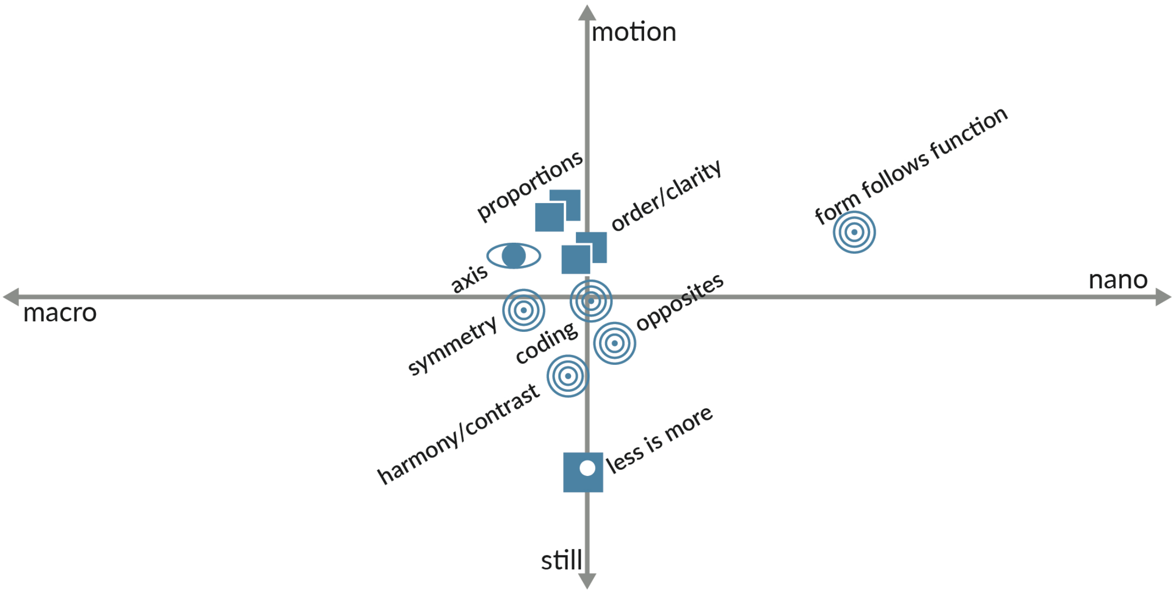

Figure S2: General design principles

The general aspects of visual communication are basically universal to any design process. It is therefore not surprising that they are basically neutral for their relevance in the macro-nano-scale and for both still and motion pictures. Also, they are equally relevant in the 2D and the 3D world. For a set of them, the communication objective is the driver for the decision: the form/structure determines the function, one way is to chose the opposite, for example harmony/symmetry or contrast/asymmetry. The overall objective needs to be clarity: chose one aspect and then make it as clear as possible. For example: chose identical size or make them really different (proportions). This also establishes a relation between the items and the eye is guided by the axes they form. The overall goals for creating attention is simplicity and visual coding is used for recognition, which makes communication more efficient and even causes the brain to complete items that are not even completely visible. Two items shift away from the center of the neutral relevance zone, but not completely out of it: Form follows function associated with the biomolecule world relates to: structure determines function, a fundamental principle of biology. As this is so important for molecular movies, it is also more important in the nano-world in general. Simplification (less is more) however is more relevant in a still image. In a motion picture, movement of one item will automatically draw attention which is especially striking if the other items in the frame are not moving at all. So the motion picture scene can be more crowded and still draw sufficient attention, making the principle more important in a still frame.

Results and analysis: Perception *(Wäger, 2014: 61ff, 76ff)*

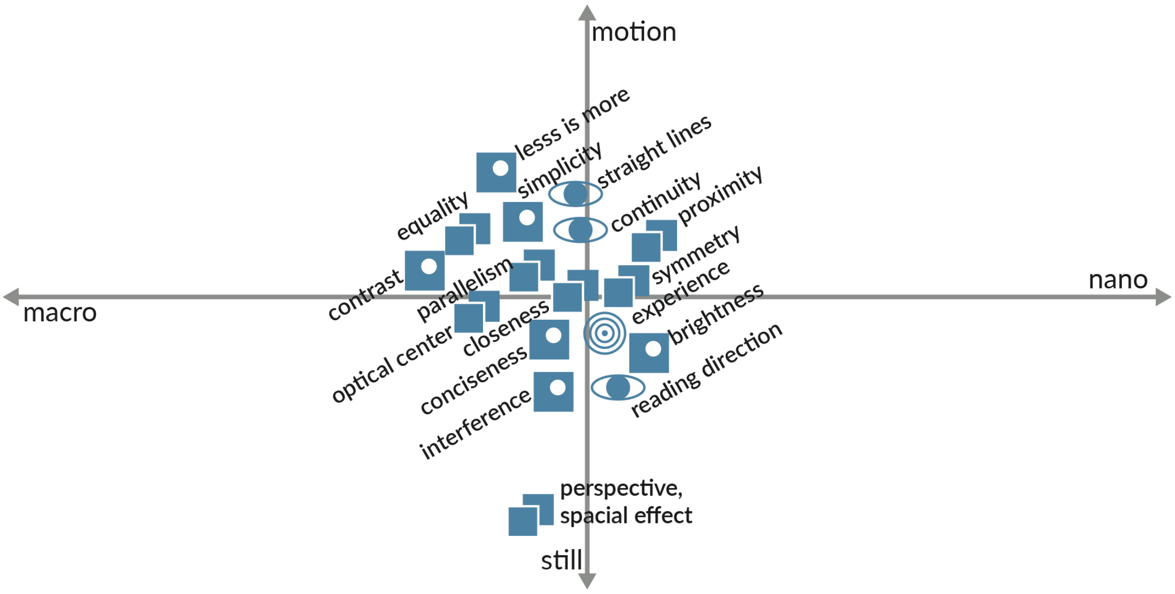

Figure S3: Perception principles

The principles on perception are also universally applied to all design processes. They too are neutral towards the scale of the content, movement and three-dimensionality, with one exception: perspective/spacial effect. Scientific visualisations may well use perspective drawings and ways to achieve a special effect. This is more relevant for data visualisation, schematics and conceptual visualisation. However, the visualisation of biological macromolecules often is very complex and only possible in tools that display them in three dimensions, adding the possibility to rotate or pan the molecule in order to grasp the spacial characteristics. Most molecular viewers and all professional animation tools therefore are actual 3D tools and the perspective and spacial impression is viewed through an equivalent of a camera perspective (or in fact a camera object in actual 3D tools). Even when we see illustrative, flat and truly 2D images of biological macromolecules they are rarely drawn (by hand or digitally) but rather renders of 3D tools with special effects, which makes this principle less relevant for 3D (in fact, perspective is given there automatically).

Results and analysis: Form and Format *(Wäger, 2014: 103ff)*

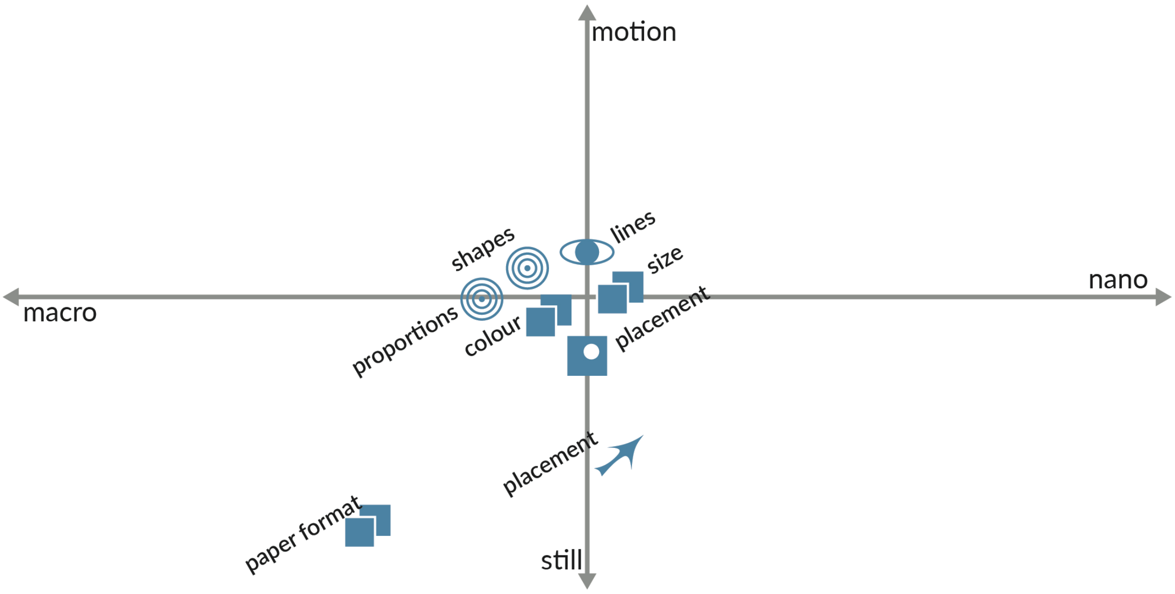

Figure S4: Form and format principles

In general, and this is also true here for the principles related to form and format, the composition of a single frame follows those universal principles and are widely neutral for all three criteria, with some smaller exceptions. Unless there is a special reason, 16:9 is the standard video format which is about the (naturally landscape) field of view of the human eyes and better suited to cover this field than the previously dominant television standard 4:3. The format therefore is of little relevance to motion pictures, but neutral to the size-dimension and 2D/3D. When it comes to forms in the frame, lines and shapes are neutral in all three criteria. Shapes in biological visualisations are predominantly organic and predetermined by nature. Nevertheless, the more detail is depicted, the stronger the pre-determination. For more abstract visualisations however, shapes may well play a role in the perception of an item. Just like the perception of perspective above, the dynamic of placement principles are most relevant in the 2D and still image world whereas they become strongly outplayed by motion in the three dimensional space. While the dynamic can be set in the first frame of a scene, it only can continue if the movement itself follows that very dynamic. Any other contradicting move will immediately have a larger effect.

The dynamic of a single frame

It is common knowledge that each motion picture is comprised of individual images (frames) that, when played at a certain frame rate, are connected by the brain and perceived as continuous movement. Usually, as low as 12 frames per seconds (fps) are sufficient to be experienced as continuous movement, standard frame rates are between 24 and 30 fps for digital screens, television or movies. Higher frame rates increase the transition-smoothness between frames and effects like motion blur can also be introduced to smooth the moving path of an object or subject. Nevertheless, the design principles for individual images can mostly be applied equivalently to motion pictures, simply to each frame individually. This is usually not done manually but by interpolating properties between keyframes, like position

and focal length of the camera, position, rotation and scale of objects, etc. This does not make principles invalid that predominantly deal with the dynamic in a single image (e.g. form > dynamic of placement), especially for the initial frame composition. However, actual movement of items will quickly overturn the initial (still) dynamic (unless the movement continues the dynamic as such).

Results and analysis: Colour *(Wäger, 2014: 177ff)*

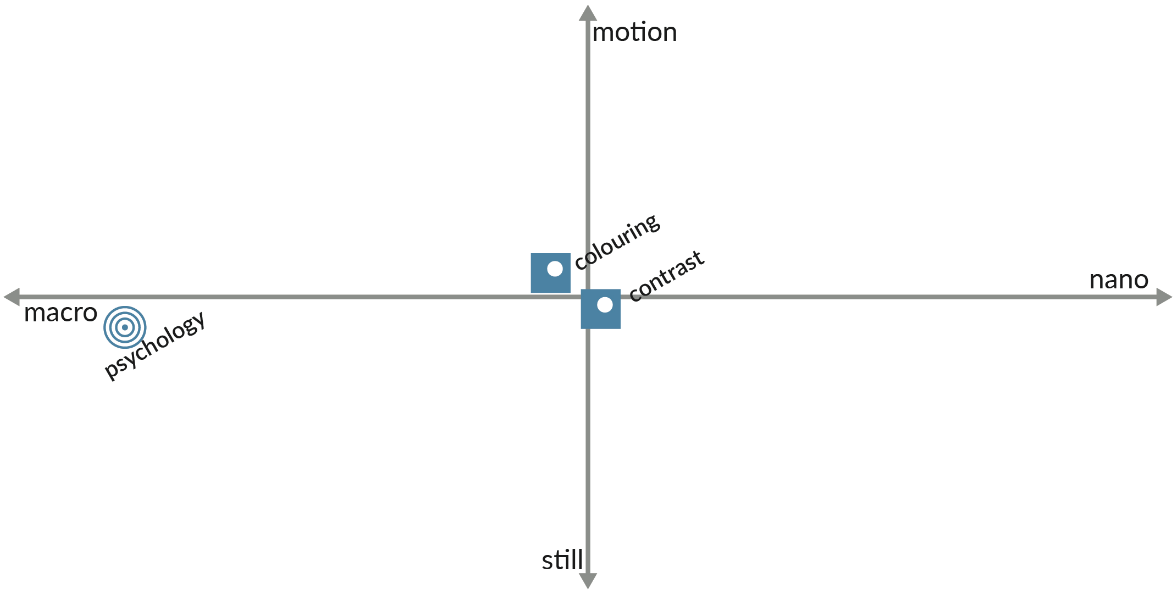

Figure S5: Colour principles

Colouring principles and contrasts are also neutral for all three criteria. However, the choice of colour and the psychological attachment to a colour has little relevance in the nano-scale biological world. The actual colour of molecules is rarely any colour at all (mostly white) or determined by some naturally occurring metal ions like iron in haemoglobin that makes blood red. Red is in fact the dominant colour of biomedical systems, as blood vessels infuse tissue like muscles; other tissue, like fat, is close to white. In fauna, the green of chlorophyll is the dominant colour. As a consequence, we are basically free to colour the components in scientific illustrations and animations based on design principles and use contrasts for emphasis. The perception of the macro-world does not have an equivalent in the nano-world, so the common psychological meanings / associations with certain colours tend not to play a role at all. Red for example, while it often stands out, is not really to be perceived as an alarm colour as red is basically the standard in the biomedical world. Nevertheless, many research fields have found some conventions to ease their communication. For example, colours of protein domains tend to be used the same way once someone has introduced an initial set. Differing from this convention would cause irritation, misunderstanding and miscommunication and is therefore avoided. However, while those conventions may restrict the use of different colour tones, there are usually still many contrast opportunities that can be used as well as colour harmonies to be generated.

Results and analysis: Interface Design *(material.io/Goolgle, 2021)*

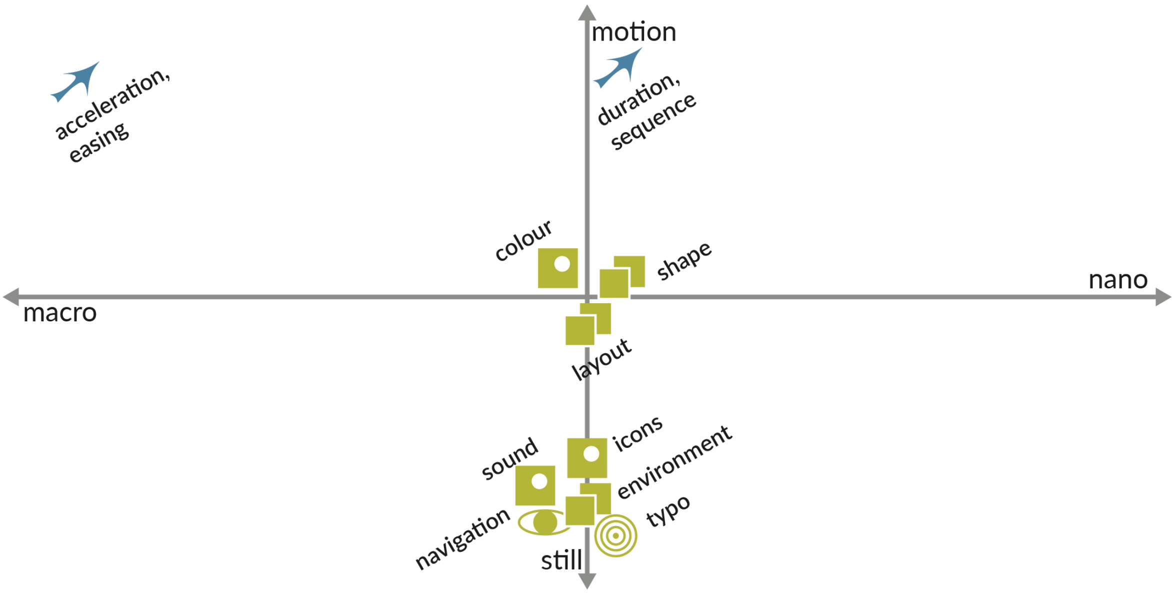

Figure S6: Interface design principles

While meant for the development of user interfaces of apps, the principles and strategies of the MATERIAL system are worth a look as well, especially the motion related parts. The actual name-giving material part, that takes the physical world as inspiration and looks at textures, light and shadow behaviour, is less relevant as they do not play the same role in the nano-world. In the macro-world, material require surfaces, whereas in the nano-world, surfaces are used as representation of some other property (like volume, charge, etc.) and is not really a physical item. For post-production and compositing, those principles are again important. The principles related to layout, colour and shape are widely consistent with the general principles above and therefore also neutral to the three criteria. On the other hand, the principles on environment, navigation, typography, sound and iconography are quite specific for user interface design and therefore not relevant for the nano-world. The principles for motion however are indeed relevant for motion pictures, but not so much for still images. In order to draw attention to a moving item, the time-in-frame (duration) needs to be appropriate. For guiding the eye, the sequence of moving items and therefore the order of events shall follow the principles. Both (duration and sequence) are therefore neutral towards the size-dimension of the system. However, this is not true for the acceleration easing. This is intended to reflect the real (macro) world but lacks an equivalent in the nano-world and is therefore not relevant in the latter.

Results and analysis: Disney Animation Principles *(Thomas and Johnston, 1981)*

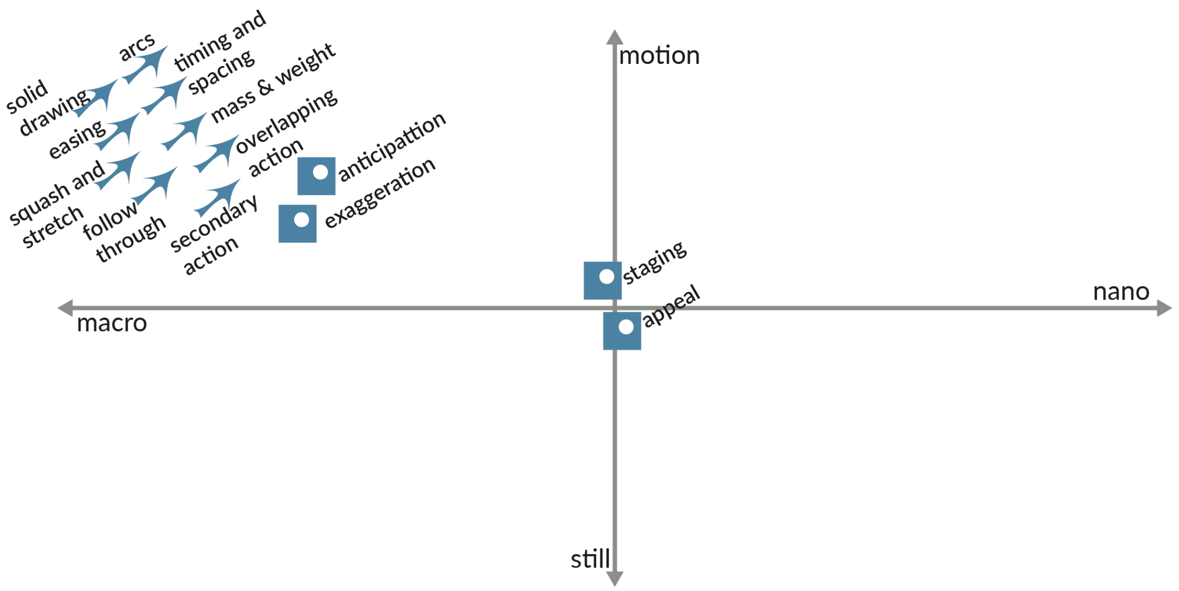

Figure S7: Disney animation principles

Most of the common animation principles that go back to the famous Disney rules are meant to recreate natural movement of characters and can be translated to macroscopic (large) items. The way they behave is supposed to follow the classical laws of mechanics and are based on our every day experience. Most people have some sort of experience for movement on the macro level and can conclude the motion pattern from the material properties. For example, we expect a tennis ball to make many large bounces and a bowling ball to make very few or no bounces at all. However, we do not have the same experience level when it comes to the micro level (e.g. the movement of a cell) and almost none on the nano level, the level of molecules and atoms. This is in fact comparable with the differences between the physical disciplines classical mechanics and quantum mechanics. We do not need to go into quantum mechanical properties of atoms and chemical bonds unless we look at specific (bio)chemical reactions explicitly. However, the lack of experience with nano-size items and movements and the very fact that the classical physical laws do not apply in the same way is sufficient to categorise all but two of the above animation principles as irrelevant in the nano-world. They are also naturally irrelevant for still images too.
In summary, molecules simply do not move in the same way as macro-objects and the attempt to recreate their natural behaviour is not compatible with the principles for macro-objects. In fact, pretending that the rules would still apply would lead to misconceptions of movement in the nano-world and they therefore should not be used. Molecules in general move through diffusion, by collisions with other molecules and based on thermal motion. This means that there is no directed force pulling or pushing two reaction partners towards each other, they basically meet by chance. As a consequence, the concepts of easing, squash and stretch, follow through, overlapping action, secondary action, arcs, anticipation and exaggeration do not have an equivalent in the nano-world. They in fact contradict the principles of scientific visualisation. Two of them however seem not far away from the behaviour of biological macromolecules. Squash and stretch may be comparable to protein

“breathing” (the internal motion of a protein due to thermal motion and small conformational changes) and follow-through to domain-domain flexibility (movement of one protein domain relative to another by flexible linkers). Stil, due to the absence of external forces causing a directed, specific movement in a protein, the two principles are considered also irrelevant in the nano-world. In many real world animations we still see linear, directed motions of two reaction partners towards each other. Even though this is not reflecting the actual scientific situation, it is often used as simplification.
Only two principles, staging and appeal, are neutral to all three categories due to their more or less generic nature.

Results and analysis: Cinematography *(Sijll, 2005; Mercado, 2010; Raschke, 2013)*

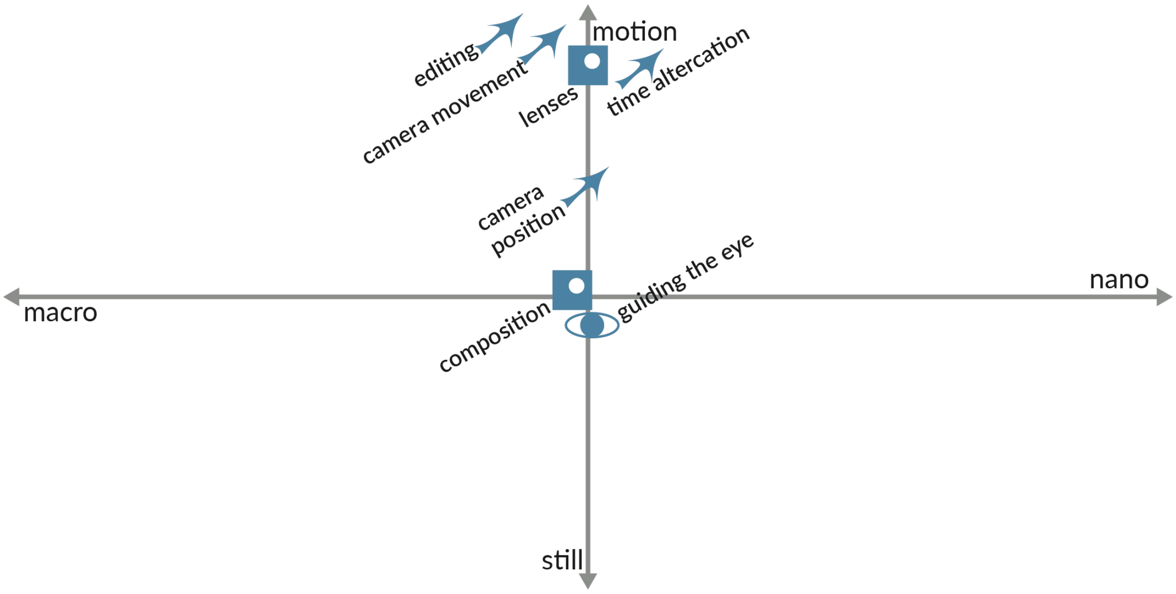

Figure S8: Cinematography principles

Two of the principle categories, composition and guiding the eye, strongly remind of the general design principles described in detail above. They are universally applicable and neutral to the three categories. The rest of the aspects is more specific for motion pictures and not relevant for still images, even though the camera perspective principles may also be applicable for a still image. They are all equally relevant in the macro and the nano-world. The understanding of character and subject may be different from actual people, but the cinematic storytelling principles still apply. They can be used in order to communicate a specific aspect of an animation.

SUMMARY

Many design principles apply universally to all design processes. They serve to draw attention, guide the eye, establish a relationship between items, convey dynamics or trigger emotions or reactions. With relation to molecular animations in three dimensions, there are some (groups of) principles that simply do not apply, mostly those that are made for two dimensions or still images. Those are the principles that convey perspective in a still image, dynamic in an image, describe the influence of the (paper) format, a large part of the user interface design principles (environment, navigation, typography, sound, iconography). They do not apply to molecular animations, but they also do not contradict the principles of scientific visualisation and therefore are not further discussed. A complete set of principles derived from (character) animation and motion graphics in fact contradicts principles of the nano-world, because the dynamic of motion is different from what most of us experience in every day life and what is described by classical mechanics/physics. Biological macromolecules in a cellular environment move differently and there is no every-day experience. The same can be said on the psychology of colours, whereas the use of colour in fact is restricted by many conventions in scientific visualisation.

Principles Relevant for Molecular Animations

The sections above give us the design principles that are relevant for molecular animations. They are the principles relevant in the nano-world and for motion pictures, or at least neutral to the given categories. Or in graphical terms: all principles of the upper right quadrant of the Figures above, including the neutral ones on or near the axes. They are listed and a selection visualised below, ordered by principle objectives (with the design area and principle title in blue italics):

general - less is more

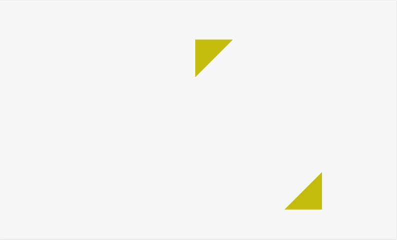

simplification for clarity, prone to interpretation

perception - simplicity

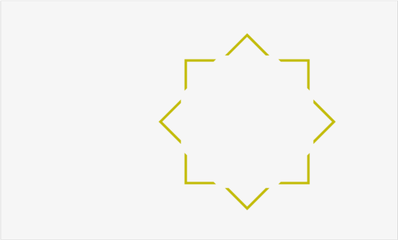

we see the easiest, obvious interpretation

perception - conciseness

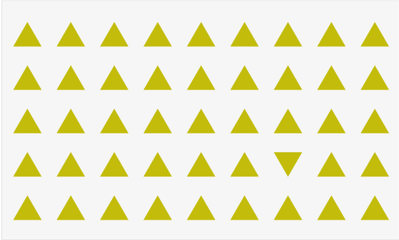

contrasting object stands out

perception - brightness

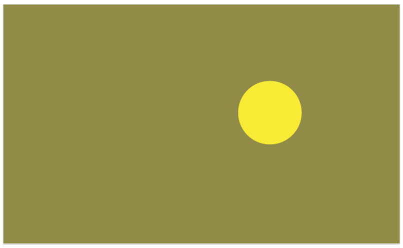

object appears bigger

perception - contrast

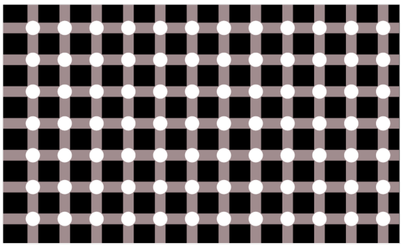

readability, too much causes flicker

perception - interference

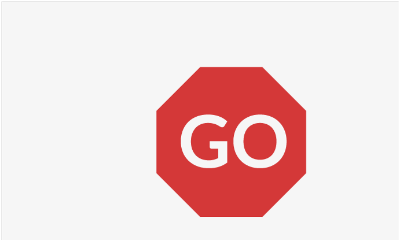

mixed messages cause irritation

form - proportion and ratios

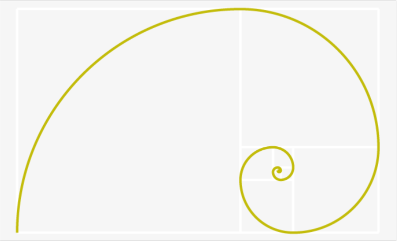

golden ratio is perceived as harmonic

form - placement

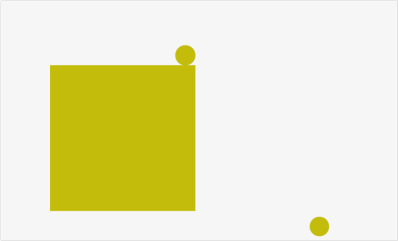

optically heavy objects offer hold for ligher

colour - contrasts

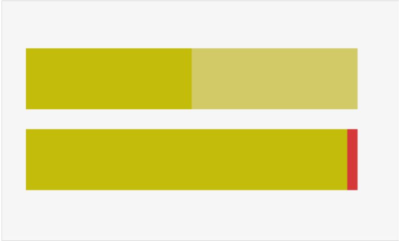

examples: quality (top), quantity (bottom)

also relevant

film/editing - assembly

assembly of shots with transitions direccts emotional response

film/camera lens - close up

move the viewer close to the detail, strong emotional connection (see also B)

film/time altercation- freeze frame

special emphasis on a frame, protected from time

film/camera movement- static

movements and changes in the frame can be noticed much better (see also B)

Figure S9: Draw attention

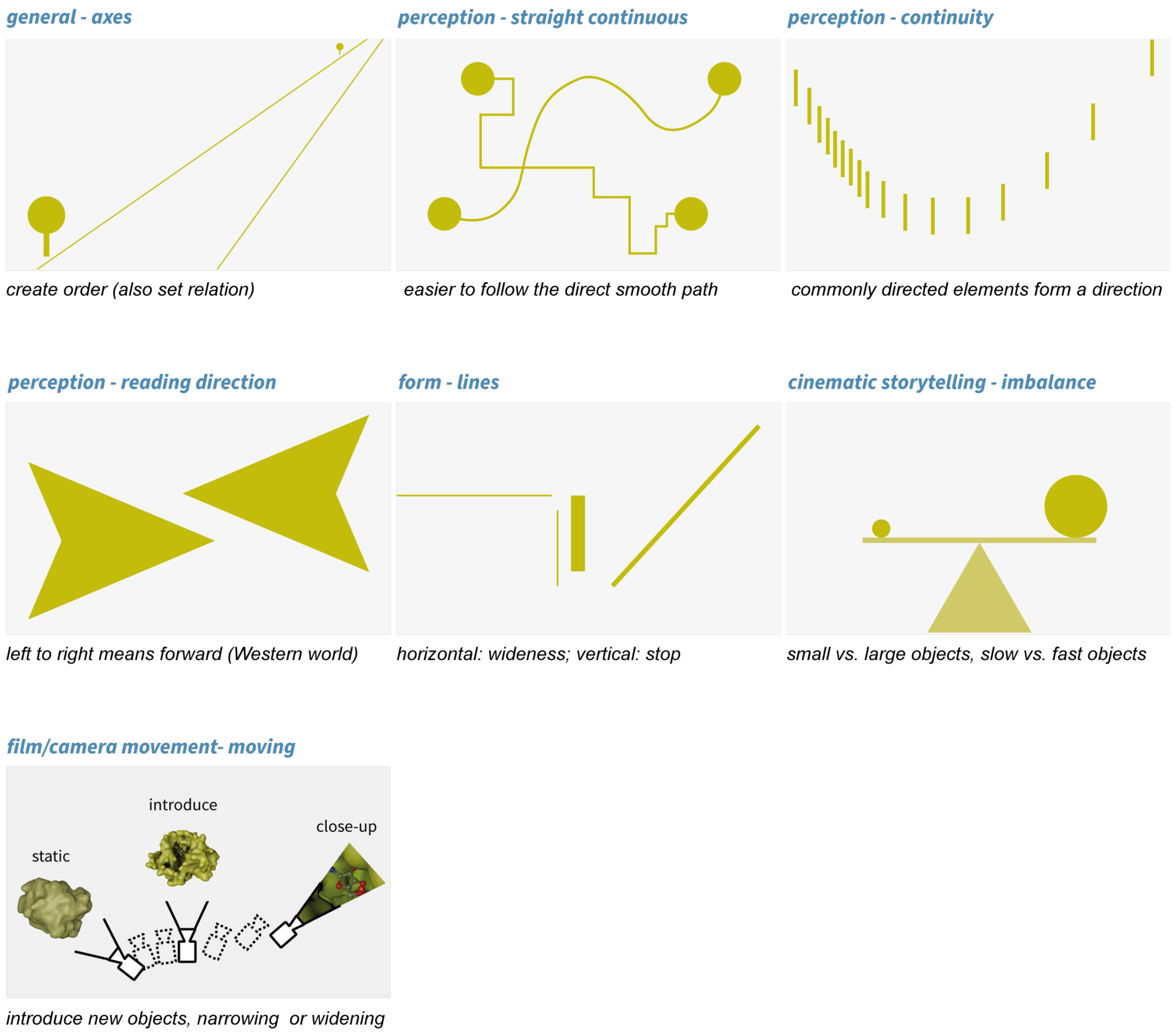

Figure S10: Guide the eye

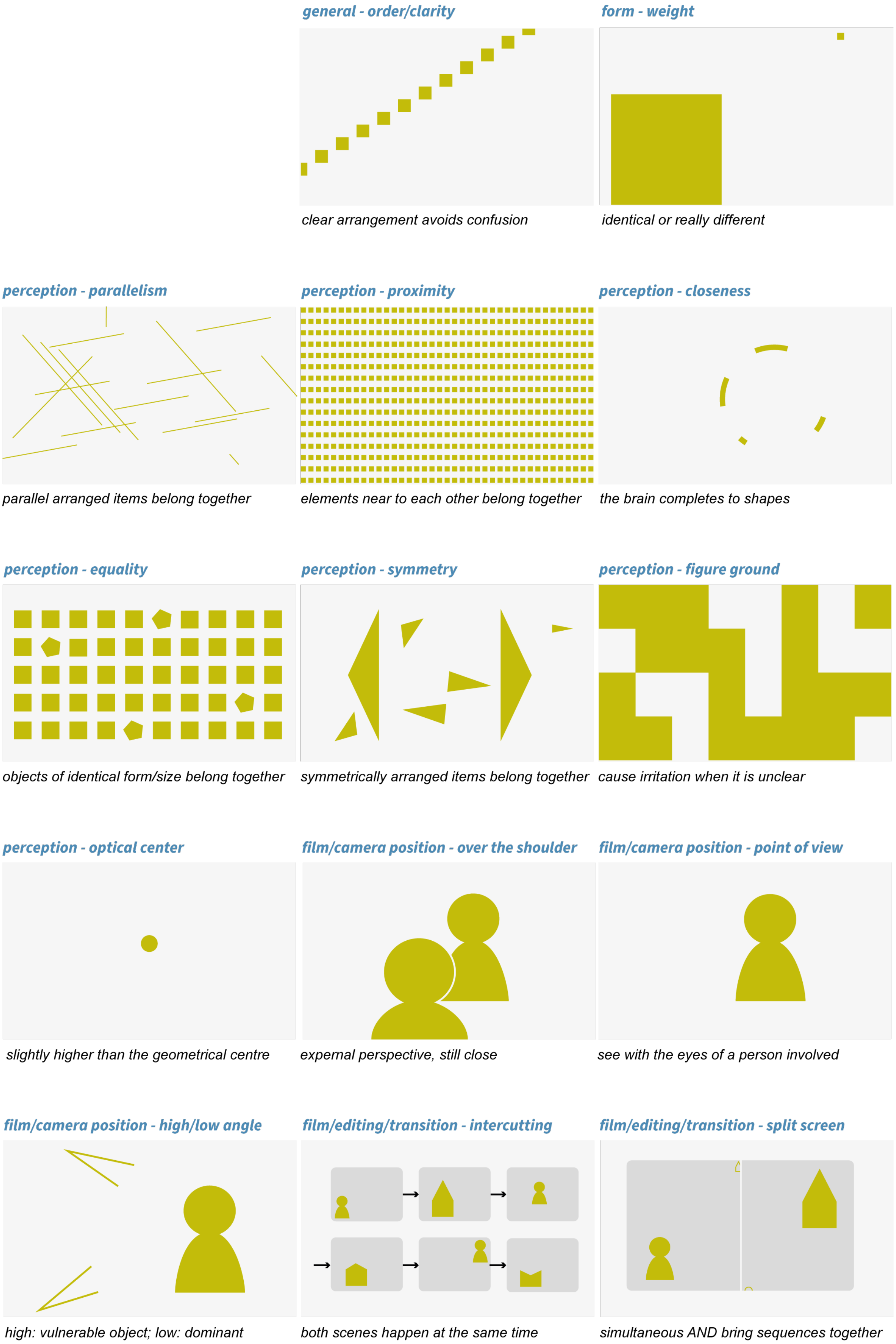

Figure S11: Relationship between items

interface design - motion duration, motion sequence

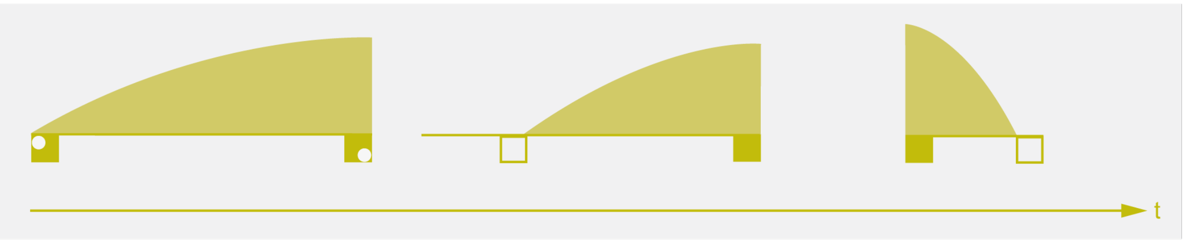

path movement

fade-in

fade-out

cinematic storytelling - composition in 3D

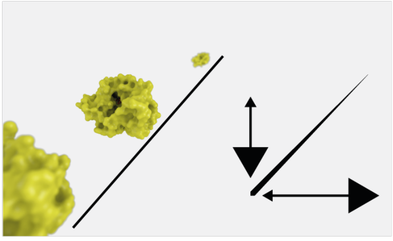

axis, up/down, left/right, depth of field

film - time altercation

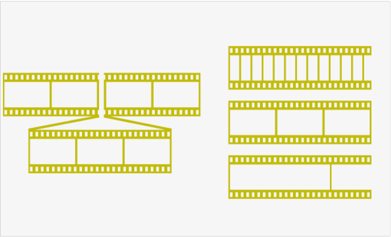

left: flashback/-forward; right: time-lapse (top), real time (middle), slow motion (bottom)

also relevant

film/editing/transition -

montage: series of quick cuts, cover long period of time, disconnected scenes connect to the bigger picture

one-take: no cuts, real-time experience

matching transition: visually match to convey continuity or contract

Figure S12: Convey dynamics

general - form follows function

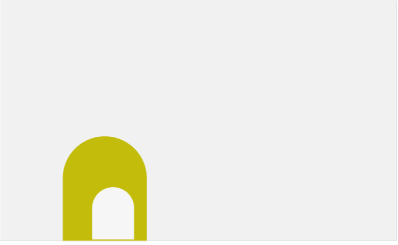

balance of function and aesthetics

general - visual coding

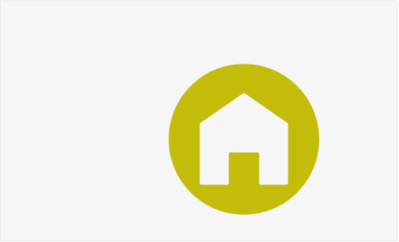

icons create understanding

general - harmony/contrast

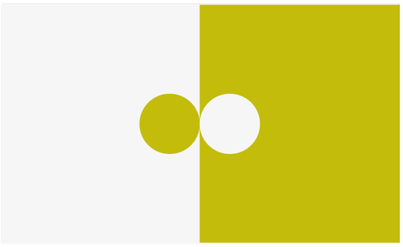

harmony seems calm, contrast striking

general - (a)symmetry

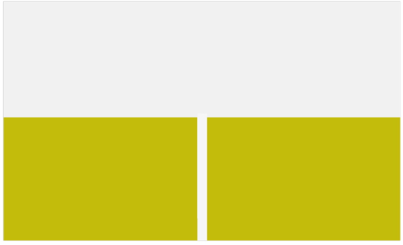

symmetry static, asymmetry dynamic

perception - experience

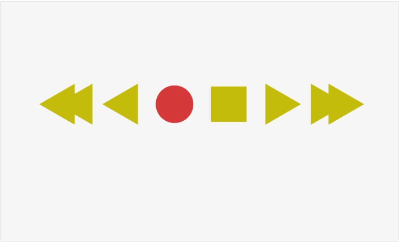

conventions for recognition

also relevant

film/editing/transition - smash cut

sudden, unexpected change, disconnect scenes

film/camera lens - different medium

influences the perception, for example distortion by window, mystery by dust, lack of gravity underwater

film/camera lens - wide/tele

wide for vistas, convey distance; tele for focus on object, separates from backgound

Figure S13: Create emotions/reactions

Principles of Scientific Visualisation and Animation

The nano-world of scientific visualisation has developed own rules on top of the general, universal design principles, just like many other design disciplines have their own set of rules. They are the basis for the development of the guidelines in the main article and summarised below in keywords.

Principles NOT relevant for molecular animation

The following principles are not further considered because they apply to still images or extended reality which both are not at the focus of this work.

- render style: shading (object boundaries, non-photorealistic renders);
- render style: stereo images;
- dynamic in images: all methods;
- responsiveness - only relevant for interaction design :
- resolution over immersion - only relevant for mixed reality media, e.g. virtual reality, augmented reality.

Principles relevant for molecular animation

General Visualisation Principles (Munzner, 2014: 117ff)

- no unjustified 3D (2D): a decision to be made at the beginning or for each scene;
- eyes beat memory: dependent on the communication tool and engagement time;
- overview first, zoom and filter, details on demand: depending on the communication tool and the narrative;
- effective use of colour: a general question of the design and a case by case decision that is often restricted by conventions.;
- function first, form next: somehow a non-principle, because it should always be the goal to achieve both effectivity and beauty and everybody does what he/she can.

Abstraction and simplification

One of the central issues of scientific visualisation and animation that is dependent on the communication objective, the audience knowledge level and the experimental data available and also a central question for the selection of an appropriate representation of a biological macromolecule. Directly connected:

Use of artistic license:

Almost always required, the amount of artistic license depends on many factors and should be handled transparently.

Advance the audience:

In fact the very reason to explain something in a scientific communication process in the first place. It is important to avoid misconceptions to maintain authenticity.

Render style

- lighting: the absence of aqueous effects needs to be respected ;
- shading: phong shading and ambient occlusion to improve the depth perception;
- clipping plane: to unveil an otherwise covered part;
- depth cueing: to draw attention or to guide the eye;
- camera angle, viewpoint: to set relations of items;
- highlighting: to draw attention or to guide the eye.

Dynamic realism

The principles (Jantzen, 2017) improve the realism of an animation and can be grouped:

- #01/03/04 - Permanent Brownian motion causes collisions and therefore movement, there are no long-range forces.
- #02/11/(10) - Biological macromolecules underlay internal flexibility but they have defined boundaries.
- #05/06/07/12 - In the cell, there are many instances of a molecule and not all react.
- #08/09 - The cell is a crowded environment that does not show aqueous effects.

Storytelling

Like in a non-fiction documentary, the issue is to find the story, a narrative within the facts that engages the viewer.
